# Dietary Strategies for the Treatment of Cadmium and Lead Toxicity

**DOI:** 10.3390/nu7010552

**Published:** 2014-01-14

**Authors:** Qixiao Zhai, Arjan Narbad, Wei Chen

**Affiliations:** 1 State Key Laboratory of Food Science and Technology, School of Food Science and Technology, Jiangnan University, 1800 LiHu Road, Wuxi 214122, China; E-Mail: zhaiqixiao@sina.com; 2 Gut Health and Food Safety Programme, Institute of Food Research, Norwich NR4 7UA, UK; E-Mail: arjan.narbad@ifr.ac.uk; 3 Synergistic Innovation Center for Food Safety and Nutrition, Wuxi 214122, China

**Keywords:** dietary supplement, heavy metal, essential metals, vitamins, edible plants, phytochemicals, probiotics

## Abstract

Cadmium (Cd) and lead (Pb) are toxic heavy metals that cause adverse health effects in humans and animals. Chelation therapy, the conventional treatment for heavy metal toxicity, is reported to have a number of safety and efficacy issues. Recent studies have shown that dietary supplements play important roles in protecting against Cd and Pb toxicity. This paper reviews the evidence for protective effects of essential metals, vitamins, edible plants, phytochemicals, probiotics and other dietary supplements against Cd and Pb toxicity and describes the proposed possible mechanisms. Based on these findings, dietary strategies are recommended for people at risk of Cd and Pb exposure. The application of these strategies is advantageous for both the prevention and alleviation of Cd and Pb toxicity, as such supplements can be added easily and affordably to the daily diet and are expected to have very few side effects compared to the chelation therapy.

## 1. Introduction

Heavy metal toxicity is one of the oldest environmental problems and remains a serious health concern today. Cadmium (Cd) and lead (Pb) are common toxic heavy metals in the environment. The general public is exposed to Cd and Pb through the ambient air, drinking water, food, industrial materials and consumer products [1,2]. Today, it is the developing countries that are facing the most serious Cd and Pb pollution problems. The threshold for blood lead level (BLL) thought to cause toxicity in children was 60 μg/dL in 1960s but this value was lowered to 10 μg/dL in 1991, subsequently the Centers for Disease Control and Prevention in US reported that they no longer consider any blood lead level to be safe for children [3]. As a consequence of pollution, the blood lead analyses of 15,727, 14,737 and 13,584 Chinese children in 2004, 2005, and 2006, respectively, showed 10.10%, 7.78% and 7.30% of children had BLL above 10 μg/dL [4]. A study conducted in Pb polluted areas of Egypt between 2007 and 2008 indicated that 44% of tested children had BLL above 10 μg/dL, and 37% of these had cognitive dysfunction [5]. As reported in 2010, the average BLL of Indian children from a polluted village was 15.11 ± 5.62 μg/dL [6]. The average Cd concentration of rice from polluted areas in Jiangxi Province of China was 0.59 mg/kg in 2006, which is 2.5 times higher than it was in 1987 and significantly higher than the Chinese Hygienic Standard for rice (0.20 mg/kg) [7]. A study conducted in a heavy metal polluted village in Vietnam in 2007 showed that the Cd concentration of rice was 0.31 mg/kg, significantly higher than the maximum allowable concentration for Cd in rice (0.20 mg/kg), as published by the Vietnamese Ministry of Health [8].

Cd and Pb exposure cause a broad range of adverse health effects in humans and animals. Cd toxicity is associated with pulmonary [9], renal [10], hepatic [11], skeletal [12], reproductive [13] and cardiovascular dysfunctions [14]. This non-essential metal is also classified as a group I human carcinogen by the International Agency for Research on Cancer [15]. Pb exposure induces neurologic and haematological dysfunctions [16,17], renal and hepatic damage [18,19] as well as reproductive disorders [20] in the human body. Children are especially at greater risk because they have higher intestinal Pb absorption and more vulnerable nervous systems which are still under development [16,21,22]. Although a number of different routes by which Cd and Pb cause toxicity have been reported, the underlying basic mechanisms can be summarized as the interactions between Cd/Pb and essential metals [22,23] and the oxidative stress caused by Cd/Pb exposure [24,25]. To some extent these two mechanisms are still interrelated because the metabolic disorder of essential metals such as zinc and selenium also induces adverse effects in the oxidative and antioxidative systems [26,27].

The most commonly used therapeutic strategy for heavy metal poisoning is chelation therapy to promote metal excretion. However, chelators for Cd and Pb toxicity are themselves reported to have a number of different safety and efficacy concerns. None of the chelation therapies for Cd toxicity have yet been approved for clinical use thus far [2,28]. Chelators such as CaNa_2_EDTA and meso-2,3-dimercaptosuccinic acid (DMSA) have been reported to have protective effects against Pb toxicity. However, CaNa_2_EDTA can cause renal toxicity (at the proximal tubule particularly), especially during repeated high doses treatment (above 75 mg/kg) and in subjects with previous history of kidney damage [29]. Because of its relative lack of specificity, other essential metals such as zinc, iron and manganese are also reported to be excreted and depleted following CaNa_2_EDTA therapy [30]. DMSA also has side effects such as appetite loss, nausea and diarrhea [31]. A study of children being treated with DMSA showed that 12% had mild gastrointestinal symptoms and 5% experienced general malaise [32]. The development of safe and efficient strategies against Cd and Pb toxicity is therefore an area of ongoing research. Dietary supplements have been reported to play important roles in the alleviation or prevention of Cd and Pb toxicity. Dietary strategies are advantageous, as nutritional ingredients can easily and affordably be added to the daily diet and can overcome the negative side effects of the chelation therapy. 

Herein we review the potential dietary strategies for Cd and Pb toxicity of essential metal, vitamin, edible plant and dietary phytochemical supplementation and probiotics, among others. 

## 2. Essential Metals

Many studies in both animals and humans have shown that a deficiency in essential metals such as zinc [33], calcium [34] or iron [35] can lead to greater absorption and toxicity of Cd and Pb. Therefore it is logical to suggest that the supplementation with essential metals can provide protective effects against Cd and Pb intoxication. A selection of such studies listed in Table 1 show the benefits of essential metals in this context.

Zinc is one of the most well studied essential metals for the alleviation of heavy metal toxicity. As zinc has similar chemical and physical properties to Cd and Pb, it competes for the binding sites of metal absorptive and enzymatic proteins [36]. Intake of zinc also induces the synthesis of metallothionein (MT) [37], a low molecular weight protein that has a high affinity for Cd and causes detoxification by binding Cd [38]. Zinc supplementation effectively protects the activity of blood δ-aminolevulinic acid dehydratase (ALAD), a zinc-dependent enzyme that is very sensitive to Pb toxicity [39]. Moreover, zinc intake has been reported to alleviate the oxidative stress caused by Cd and Pb exposure [40,41], which may be due to zinc’s functionality as a cofactor of the antioxidant enzyme copper zinc-superoxide dismutase (Cu/Zn SOD). 

A considerable number of studies have shown that selenium administration is protective against Cd and Pb toxicity within a range of different organs of mice including the brain, lungs, liver, kidneys and blood. Selenium is a cofactor of the antioxidant enzyme glutathione peroxidase (GPx) and it contributes to the antioxidant defence system, which enables it to alleviate Cd and Pb toxicity by reducing the Cd/Pb-induced oxidative stress and enhancing the antioxidant capacity of the host [42,43]. It is also believed that selenium may form inactive complexes with heavy metals which can further enhance their detoxification [44].

Iron competes with Cd for access to intestinal metal uptake transporters including divalent metal transporter-1 (DMT1) and metal transporter protein 1 (MTP1), which may explain the decrease in intestinal Cd absorption after iron supplementation [45]. Moreover, the expression of these transporters is often modulated by nutritional status of essential minerals such as iron and zinc [28]. For instance iron deficiency has been reported to up-regulate the expression of DMT1 in intestinal epithelium [45,46]. Hence iron supplement can prevent or limit Cd absorption by reducing the expression of such transporters. On the other hand, as iron is a component of the heme complex, the deficiency of iron will enhance Pb toxicity to the heme synthesis system [47]. Other essential metals, such as calcium and magnesium, have also been reported to be effective against Cd and Pb toxicity (Table 1). These essential metals can reduce the heavy metal burden by competing with Pb or Cd for intestinal absorption and prevent heavy metal induced tissue damage by competitive binding to active sites of the enzymes [48,49].

**Table 1:** Selected studies on the protective effects of essential metals against Cd and Pb toxicity.

Essential Metal	Administered Form	Duration	AnimalModel	TargetSites	ProtectiveEffects	Ref.
Zinc	40 mg/L ZnCl_2_ in drinking water	30 days	Male rats exposed to 40 mg/L CdCl_2_ in drinking water	Testes	Zinc restored the activity of GPx and SOD in the testes and attenuated DNA oxidation in the gonads.	[40]
	0.02% Zn^2+^ in drinking water	PND 1 to PND 21, stop at weaning	Pregnant mice exposed to 0.2% Pb-acetate in drinking water	Brain	Zinc restored the activity of SOD, XO and CAT, and decreased the LP levels in the pups’ brains.	[41]
Selenium	20 μmol/kg b.w. (PhSe)_2 _by oral treatment	4 weeks	Male rats exposed to 10 μmol/kg b.w. CdCl_2_ (s.c.)	Brain and lungs	(PhSe)_2_ restored the activity of SOD and CAT, increased the vitamin C content and decreased the level of LP in the brain. It also decreased the Cd level in the lungs.	[42]
	0.2 mg/L Na_2_SeO_3_ in drinking water	21 days	Lactating rats exposed to 100 mg/L Pb-acetate in drinking water	Brain and nervous system	Na_2_SeO_3_ improved the spatial memory and the level of LTP and decreased neuron apoptosis in the pups.	[43]
Iron	120 mg/kg b.w. Fe in diet	4 or 8 weeks	Male rats exposed to 100 μg/kg b.w. CdCl_2_ by oral gavage	Kidney, liver and intestinal tract	An iron-sufficient diet decreased the Cd burden in the tissue and regulated intestinal Cd absorption through the iron transporters.	[45]
Calcium	0.02% Ca^2+^ in drinking water	GD 6 to PND 21	Pregnant mice exposed to 0.2% Pb-acetate in drinking water	Brain and nervous system	Calcium decreased the synaptosomal AChE and mitochondrial MAO activity and improved the pups’ total locomotor activity and exploratory behaviour.	[48]
Magnesium	20 mg/kg b.w. Mg orally	1 or 2 weeks	Male mice exposed to 10 mg/kg b.w. Cd	Testes and kidneys	Mg pre-treatment was efficient in restoring the renal and testis GSH levels.	[49]

AChE, acetylcholinesterase; b.w., body weight; CAT, catalase; GD, gestational day; GPx, glutathione peroxidase; GSH, glutathione; LTP, hippocampal long-term potentiation; LP, lipid peroxidation; MAO, monoamine oxidase; PND, postnatal day; s.c., subcutaneously; SOD, superoxide dismutase; XO, xanthine oxidase.

In summary, these essential metals decrease intestinal Cd and Pb absorption, recover the essential metal homeostasis and alleviate the oxidative stress caused by Cd and Pb toxicity. Diet associated essential metal supplementation should be regarded as important for essential metal-deficient people, such as children and pregnant women. Because without sufficient essential metal stores to prevent heavy metal absorption, these people are especially susceptible to heavy metal toxicity [16,21,50,51]. It should also be noted that Cd and Pb exposure cause the loss of essential metals, which leads to complications such as iron-deficiency anaemia and osteoporosis [52,53]. Appropriate concentrations of essential metal supplementation is therefore also beneficial for preventing these complications. 

## 3. Vitamins

Vitamins are vital nutrients for humans and can easily be obtained from the diet. Vitamin C, B_1_ and B_6_ deficiencies have been reported to enhance sensitivity towards Cd and Pb toxicity [54,55]. Vitamin supplementation has proved to be effective against Cd and Pb toxicity in both human and animal studies. 

Vitamins C and E are natural non-enzymatic antioxidants that are able to scavenge free radicals and decrease lipid peroxidation. Many studies on the effects of vitamins C and E on Cd and Pb intoxication have been performed. Vitamin C attenuates the oxidative damage and histopathological changes induced by CdCl_2_ in the lungs and brain of rats [56]. It has similar protective effects in the liver, kidney, brain and the testes of Pb-exposed rats [57]. Apart from its well-established antioxidant properties, vitamin C has been reported to act as a chelating agent of Pb, with a similar potency to that of EDTA [58]. Probably due to this chelating capacity, a decrease of blood Pb levels from 1.8 ± 0.05 μmol/L to 0.4 ± 0.05 μmol/L (*p* ≤ 0.01) was observed in a study of 75 adult smokers receiving 1 g vitamin C daily for one week [59]. However, it is noteworthy that very few animal studies can confirm the positive impact of vitamin C on reducing blood Pb levels. Indeed a human clinical study with 52 adult male subjects found that 3 months of vitamin C supplementation had no impact on the levels of Pb in blood or hair [60]. Pre-treatment with vitamin E exhibits protective effects against Cd toxicity, as measured by the haematological values, lipid peroxide concentration and antioxidant defence system in the blood, liver and brain of rats [61,62]. The combination of vitamins C and E also resulted in reduction of oxidative stress-related damage to spermatogenesis in Cd-exposed mice [63] and protects steroid production in Cd-exposed rats [64]. In a recent study of workers exposed to Pb (73 μg Pb/dL blood), after one year of oral vitamin C and E supplementation (1 g daily vitamin C and 400 IU daily vitamin E), lipid peroxidation in erythrocytes was reduced to values between 47.1% and 69.4%, which were no longer statistically different to those of the non-Pb exposed workers. The total antioxidant capacity in erythrocytes was also reversed to values between 58.9% and 67.7% in Pb-exposed workers after treatment, a level that was similar to those in non-Pb exposed workers [65].

Dietary vitamin B_1_ supplement has been reported to decrease Pb levels in the liver, kidneys, bone and blood, and recover ALAD activity in the blood in animal studies [66,67,68]. Vitamin B_1_ influences the absorption of Pb and its pyrimidine ring mediates its interaction with Pb, which may cause an increase in Pb excretion and the alleviation of its toxicity [68,69]. Vitamin B_6_ has also been found to be effective in reducing accumulation of Pb in tissues and in reduction of inhibition of ALAD activity. This function is likely to be attributed to the ring nitrogen atom in its structure, which can chelate Pb before it is absorbed [70].

## 4. Edible Plants and Dietary Phytochemicals

Vegetables, fruits and other edible plants are important dietary sources of vitamins and essential metals. Edible plant supplementation at sufficient levels can promote the levels of the vitamins and essential metals in the human body, which in turn can decrease the risks of Cd and Pb toxicity. Moreover, edible plants provide a great variety of other nutrients, such as dietary protein and phytochemicals, which have been reported to have beneficial effects against Cd and Pb toxicity (Table 2 and Table 3).

A selection of studies on the protective effects of edible plants against Cd and Pb toxicity is presented in Table 2. Soybean for example has been a part of the Southeast Asian diet for millennia. Two recent animal studies showed that dietary soybean supplementation helped to prevent arterial and cardiac injury by alleviating the oxidative stress induced by Cd toxicity [71,72]. The authors suggested that the soybean protein and soybean isoflavones provided the observed antioxidant effects. 

Garlic, ginger and onion are used as ingredients for flavour, aroma and taste enhancement all over the world. Garlic is also a well known medicinal plant. Garlic extract alleviates Pb-induced neural, hepatic, renal and haematic toxicity in rats and protects against Cd-induced mitochondrial injury and apoptosis in tissue culture models [73,74,75,76]. Based on these studies, garlic’s protective property against Cd and Pb toxicity can be attributed to (1) its antioxidative ability, provided by organo-sulphur compounds such as diallyl tetrasulfide; (2) its chelation ability, provided by sulphur-containing amino acids and compounds with free carboxyl and amino groups, which in turn promotes the excretion of Pb or Cd from the body; and (3) the prevention of Cd and Pb intestinal absorption, by its sulphur-containing amino acids such as S-allyl cysteine and S-allyl mercaptocysteine. Ginger and onion have similar antioxidant capacities to garlic, and supplementation with these food ingredients gave protection against Pb-induced renal and developmental toxicity and Cd-induced gonadotoxic and spermiotoxic effects in rats [77,78,79]. 

Green tea and curry leaves are commonly used in Asian cooking and are endowed with numerous potential benefits to human health including alleviating the oxidative stress induced in diabetes [80] and protecting liver from ethanol induced toxicity [81]. These plants are also gaining popularity in the West. The protective effect of green tea against Cd and Pb toxicity is mainly due to its active constituent, catechins, which are discussed later in this section. The flavonoids and phenols in curry leaves can function as antioxidants and as potential chelators, which offer protection against Cd-induced cardiac toxicity [82]. Fruits such as grapes are also effective against Cd toxicity [83]. Besides the function of vitamins and essential metals in grapes, the abundant polyphenols such as anthocyanins may also alleviate the oxidative stress caused by Cd and Pb toxicity. Tomato is regarded as one of the most powerful natural antioxidants [84] and can prevent renal toxicity induced by Pb exposure in rats [85]. Moreover, tomato has been reported to produce metal chelating proteins and phytochelatins when exposed to heavy metal ions [86,87]. In fact the oral administration of tomato has been shown to significantly reduce the accumulation of heavy metals (Cd, Pb and Hg) in the liver of rats [88].

**Table 2:** Selected studies on the protective effects of edible plants against Cd and Pb toxicity.

Edible Plant	Administered Form	Duration	Animal Model	Target Sites	Protective Effects	Ref.
Soybean	Diet containing soybean as a protein source	60 days	Male rats exposed to 100 mg/L CdCl_2_ in drinking water	Heart and aorta	A soybean-based diet ameliorated cardiac and aorta oxidative stress and recovered morphological alterations in the aorta.	[71,72]
Garlic (*Allium sativum*)	250 or 500 mg/kg b.w. garlic extract orally	30 days	Male mice exposed to 50 mg/kg b.w. Pb-nitrate orally	Blood, kidneys and brain	Garlic decreased the Pb burden and recovered immunological parameters in the blood and tissues.	[73]
Ginger (*Zingiber officinale*)	150 mg/kg b.w. ginger extract by oral gavage	1 or 3 weeks	Male rats exposed to 300 mg/kg b.w. Pb-nitrate by oral gavage	Kidneys	Ginger recovered the GSH level and the activity of antioxidant enzymes and alleviated renal histological changes.	[77]
Onion (*Allium cepa*)	5 mL/kg b.w. onion extract by oral gavage	4 weeks	Male rats exposed to 15 mg/kg b.w. Cd	Testis	Onion reduced testicular oxidative damage and alleviated spermiotoxicity.	[78]
Green tea	1.5% w/v green tea extract in drinking water	8 weeks	Male rats exposed to 0.4% Pb-acetate in drinking water	Liver	Green tea recovered hepatic function and alleviated histological changes in the liver.	[89]
Curry leaf (*Murraya koenigii*)	100 mg/kg b.w. curry leaf extract orally	15 days	Male rats exposed to 0.44 mg/kg b.w. CdCl_2_ s.c.	Heart	Curry leaf increased the activity of cardiac antioxidant enzymes and decreased the cardiac LP and Cd levels.	[82]
Grape	1.18 or 2.36 g/kg b.w. grape juice concentrate orally	56 days	Male rats exposed to 1.2 mg/kg b.w. CdCl_2_ i.p.	Testis	Grape improved serum testosterone levels, the relative weight of the epididymis and the percentage of normal sperm.	[83]
Tomato	1.5 mL tomato paste orally	8 weeks	Male rats exposed to 1% Pb-acetate in drinking water	Kidney	Tomato intake recovered renal function and prevented the alterations of antioxidant enzymes activities in blood plasma.	[85]

b.w., body weight; GSH, glutathione; i.p., intraperitoneally; LP, lipid peroxidation; s.c., subcutaneously.

**Table 3:** Protective mechanisms of phytochemicals against Cd and Pb toxicity and their food sources.

Phytochemical	Toxic Metal	Protective Mechanisms	Ref.	Food Sources
Quercetin	Cd	Quercetin induces eNOS, iNOS, COX-2 and MT expression.	[90,91]	Onion, tomato, capers and radish
	Pb	Quercetin modulates the MAPKs and NF-κB signalling pathway and forms excretable complex with Pb.	[92,93,94]	
Catechin	Cd	Catechin inhibits Cd absorption and normalises bone metabolic disorders through the bone mineral density, bone mineral content and bone calcium content.	[95]	Tea, cocoa, peach and berries.
	Pb	Catechin protects hepatic cell membrane fluidity, increases cell viability and modulates oxidative stress.	[96]	
Anthocyanin	Cd	Anthocyanin protects against Cd-induced oxidative stress.	[97]	Cherry, grape and berries.
	Pb	Anthocyanin appears to effectively diminish oxidative stress.	[98,99]	
Curcumin	Cd	Curcumin protects against Cd-induced lipid peroxidation.	[100,101]	Turmeric
	Pb	Curcumin binds Pb to form an excretable complex, reducing neurotoxicity.	[102]	
Naringenin	Cd	Naringenin quenches free radicals, recovers antioxidant enzyme activity and chelates Cd.	[103]	Orange, grapefruit and tomato
γ-Oryzanol	Cd	γ-Oryzanol reduces the testicular Cd concentration, improves ALAD activity and prevents lipid peroxidation.	[104]	Rice
Puerarin	Pb	Puerarin modulates the PI3K/Akt/eNOS pathway, reduces reactive oxygen species and protects against DNA damage and apoptosis.	[105,106]	Pueraria

ALAD, δ-aminolevulinic acid dehydratase; Akt, protein kinase B; COX-2, cyclooxygenase-2; eNOS, endothelial nitric oxide synthase; iNOS, inducible nitric oxide synthase; MAPKs, mitogen-activated protein kinases; MT, metallothionein; NF-κB, nuclear factor kappa B; PI3K, phosphoinositide-3-kinase.

Other plants, such as ginseng (*Panax ginseng* Meyer) [107], liquorice (*Glycyrrhizae radix*) [108], torch ginger (*Etlingera elatior*) [109] and tossa jute (*Corchorus olitorius*) [110] are also reported to have protective effects against Cd and Pb toxicity. Some of these plants such as tossa jute (used as a vegetable and food ingredient common to the people of Eastern Asia and Africa) or torch ginger (used in Malaysian local dishes) are popular dietary components in certain areas, whereas the others are routinely added in in candies and beverages (such as liquorice). They can therefore be recommended as dietary supplements for the prevention and alleviation of heavy metal intoxication to populations that are at risk of heavy metal exposure and who regularly consume these plants. 

Some studies designed to explore the protective mechanisms have investigated the effects of specific plant-derived phytochemicals against Cd and Pb toxicity, rather than the intact plant itself. Table 3 presents a selection of related phytochemicals, their protective mechanisms and their food sources. Most of these phytochemicals are phenolic or isoflavone in nature and are found in commonly consumed fruit and vegetables. These bioactive compounds can act as oxygen free radical scavengers or metal chelators, which enables them to be used as natural antagonists to Cd and Pb toxicity.

## 5. Probiotics as Functional Food Supplements

Probiotics are defined as “live micro-organisms which, when administered in adequate amounts, confer a health benefit on the host” (WHO 2001). Most commercial probiotics contain species of *Bifidobacterium*, *Bacillus*, *Lactobacillus* as well as the yeast *Saccharomyces boulardii* [111]. Probiotics is now a multibillion dollar industry. There is significant number of studies indicating the benefits of probiotics in relation to antibiotic associated diarrhoea, allegy, lactose intolerance, reduction of cholesterol as well as development of immune system and protection against gut pathogens [112,113]. Some species of lactic acid bacteria (LAB) including *Lactobacillus rhamnosus*, *L. plantarum*, and *Bifidobacterium longum* are capable of binding heavy metals *in vitro* [114,115]. Moreover, LAB are known to have antioxidative properties in human subjects [116,117], which may be another important characteristic for heavy metal toxicity protection. On the basis of these functions, specific LAB have the potential to be developed as probiotics for alleviation and treatment of heavy metal toxicity. This hypothesis was also proposed in a recent review by Monachese *et al.* [118]. 

Our work has demonstrated that two lactobacilli strains exhibit protective effects against Cd and Pb toxicity in mice. *L. plantarum* CCFM8610, a probiotic with a good Cd binding capacity, is able to protect mice from acute and chronic Cd toxicity via its intestinal sequestration and antioxidant effects [119,120]. The oral administration of this strain effectively decreased intestinal Cd absorption, reduced Cd accumulation in tissue, alleviated tissue oxidative stress, reversed hepatic and renal damage, and ameliorated the corresponding histopathological changes of Cd-exposed mice. *L. plantarum* CCFM8661 protects against Pb toxicity by recovering the blood ALAD activity, decreasing the Pb levels in the blood and tissues and preventing Pb-induced oxidative stress [121]. Several recent reports confirmed that other probiotics may also be protective against heavy metal toxicity. A mixture of *L. rhamnosus* Rosell-11, *L. acidophilus* Rosell-52 and *B. longum* Rosell-175 significantly reduced Cd-induced genotoxicity both *in vitro* using liver tissue culture and in rats [122]. Another study investigated the potential of *L. rhamnosus* GR-1 supplemented yogurt to lower heavy metal levels in at-risk populations of pregnant women and in children in Tanzania [123]. Their results showed that blood levels of mercury and arsenic of pregnant women increased in the control groups (*p* < 0.05) but remained stable in the probiotic group, indicating a protective effect of *L. rhamnosus* GR-1 consumption. This means that with confirmed protection against heavy metal toxicity in animal studies, probiotics also have the potential to prevent or treat heavy metal toxicity in humans. However, it is worth pointing out that the strain *L. rhamnosus* GR-1 does not significantly reduce blood levels of Pb and Cd in pregnant women or children. These studies indicate that specific probiotic or cocktails of probiotic mixes may be required for protection against different types of heavy metal toxicity.

Lactobacilli are widely used in the food industry and are generally regarded as safe. The use of these probiotic lactobacilli can be considered a new dietary therapeutic strategy against heavy metal toxicity. 

## 6. Other Dietary Supplements

Other nutrients also have the potential to alleviate Cd and Pb-induced pathogenic effects. For example, royal jelly protects against Cd-induced genotoxicity and oxidative stress in mice, due to its antioxidant effects. Algae such as *Spirulina* and *Chlorella* can attenuate Cd or Pb toxicity in the liver, kidneys and brain of animals [124,125,126,127]. *Spirulina* also has marked anti-teratogenic effects in Cd-injected pregnant mice. Oral administration of a high dose of *Spirulina* significantly decreased the frequency of foetuses with exencephaly, micrognathia, and skeletal abnormalities induced by Cd [128]. Moreover, *Spirulina* has been reported to reduce the quantity of micronucleated polychromatic erythrocytes and micronucleated normochromatic erythrocytes in blood cells of Cd-exposed mice (both the mother and the foetus) [129]. These algae possess many dietary antioxidants, such as vitamin C, vitamin E, phycocyanobilin and carotenes, which enable them to alleviate toxic metal-induced oxidative stress [130]. 

## 7. Conclusions and Perspectives

**Figure 1:**
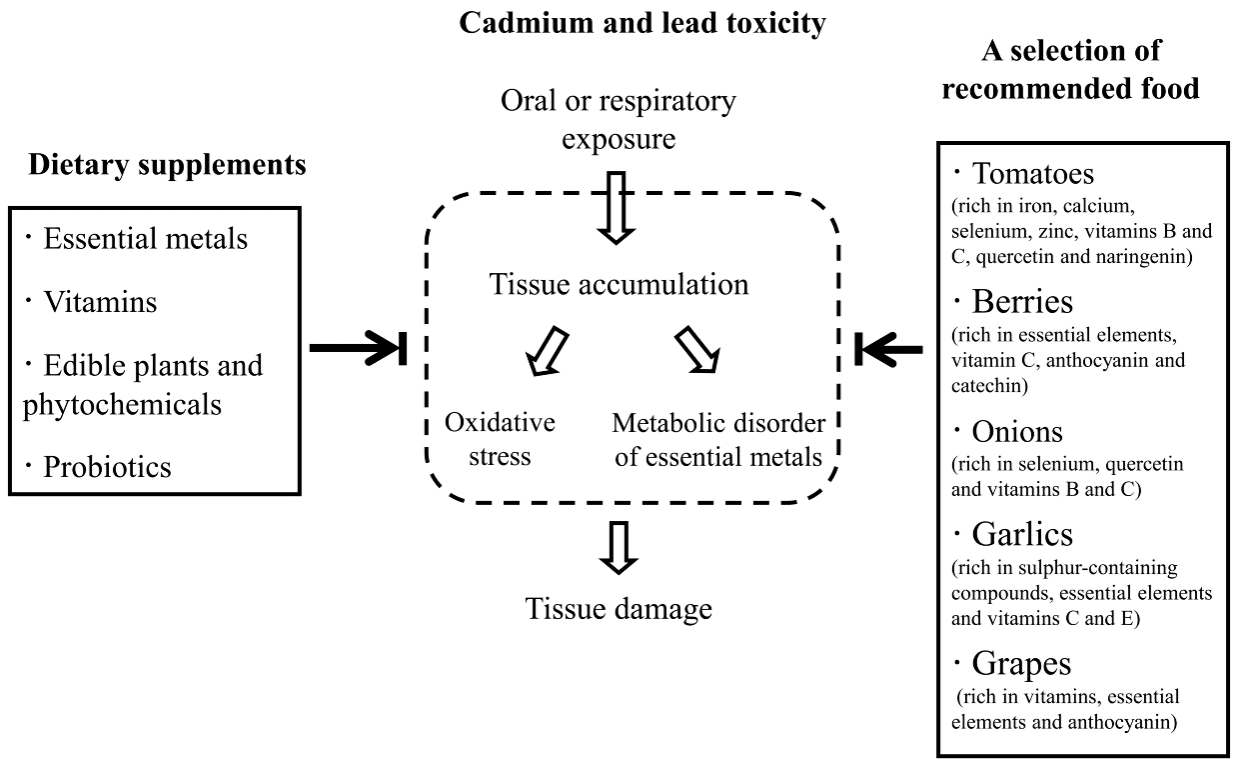
Dietary supplements and recommended strategy against cadmium and lead toxicity.

We have summarised the literature on potential dietary supplements for Cd and Pb toxicity. Based on these published reports, we recommend that people who are at risk of exposure to toxic metals ensure a sufficient intake of essential elements and vitamins and enhance their consumption of vegetables and fruit (Figure 1). Some edible plants, such as tomatoes (rich in iron, calcium, selenium, zinc, vitamins B and C, quercetin and naringenin), berries (rich in essential elements, vitamin C, anthocyanin and catechin), onions (rich in selenium, quercetin and vitamins B and C), garlics (rich in sulphur-containing compounds, essential elements and vitamins C and E) and grapes (rich in vitamins, essential elements and anthocyanin) are of special importance as natural antagonists to Cd and Pb toxicity and should be consumed on a regular basis. These dietary supplements are an affordable option, with fewer side effects than chelation therapy, for the billions of people around the world who are inadvertently exposed to toxic metals on a daily basis [118]. In addition, with the increasing contamination of the food chain, the accumulation of Cd and Pb in edible animals can present an indirect route of heavy metal poisoning in humans [1]. Therefore, providing livestock and farmed fish with the above-mentioned food interventions may also be helpful to reduce Cd and Pb exposure in humans.

While we have focused on the dietary strategies for treatment of heavy metal toxicity, intake of the suggested dietary regimes in people that are at high risk of Cd and Pb toxicity may be helpful in preventing these heavy metals from being absorbed in the body in the first place thus limiting or entirely preventing the exposure of these metals to body tissues. We need to mention that although the protective effects of essential elements, vitamins and probiotics have already been investigated in human trials, further confirmation is still necessary. It should be also noted that the studies mentioned above do not provide sufficient information on the appropriate doses of the dietary supplements in humans. It is possible that excessive consumption of essential metals, vitamins or phytochemicals may cause adverse effects in humans [65,131,132,133]. Long-duration epidemiological studies are required to determine the optimal doses of the dietary supplements, singly and in combination, to provide safe and effective dietary strategies against Cd and Pb toxicity.

## Abbreviations

AChE: acetylcholinesterase
Akt: protein kinase B
ALAD: δ-aminolevulinic acid dehydratase
BLL: blood lead level
b.w.: body weight
CAT: catalase
Cd: cadmium
COX-2: cyclooxygenase-2
DMSA: meso-2,3-dimercaptosuccinic acid
DMT1: divalent metal transporter-1
eNOS: endothelial nitric oxide synthase
GD: gestational day
GPx: glutathione peroxidase
GSH: glutathione
iNOS: inducible nitric oxide synthase
i.p.: intraperitoneally
LP: lipid peroxidation
LTP: hippocampal long-term potentiation
MAO: monoamine oxidase;
MT: metallothionein;
MTP1: metal transporter protein 1
MAPKs: mitogen-activated protein kinases
NF-κB: nuclear factor kappa B
Pb: lead
PI3K: phosphoinositide-3-kinase
PND: postnatal day
s.c.: subcutaneously
SOD: superoxide dismutase
XO: xanthine oxidase
